# Supplementary material for: Analysis of PPARγ Signaling Activity in Psoriasis
Source: Int J Mol Sci. 2021 Aug 10;22(16):8603. doi: 10.3390/ijms22168603 (PMC8395241; doi:10.3390/ijms22168603)
Supplement: Supplementary file 1 [file ijms-22-08603-s001.zip › Supplemental materials_Analysis of PPARg signaling activity in psoriasis/Pathway models/Models images and html files/PPARG inhibits phenotypes assotiated with psoriasis/104843.html]

stroke


# Disease stroke

|  |  |
| --- | --- |
| URN | urn:agi-meshdis:Stroke |
| Total Entities | 5 |
| Connectivity | 8140 |
| Name | stroke |

---

|  |  |
| --- | --- |
| ChildConcepts | brain infarction |
|  | lacunar infarction |
|  | acute stroke |
|  | heat stroke |
|  | embolic stroke |

---

|  |  |
| --- | --- |
| Pathway | Proteins with Altered Expression in Aging |
|  | Valdecoxib Induced Ischemic Disease |
|  | PPARG inhibits phenotypes assotiated with psoriasis |
|  | Neighbors of stroke |

---

|  |  |
| --- | --- |
| MedScan ID | 9000425 |

---

|  |  |
| --- | --- |
| Alias | cerebrum stroke |
|  | stroke syndromes |
|  | cerebrovascular insult |
|  | apoplexy |
|  | attack of the brain |
|  | brain insults |
|  | cerebral apoplexia |
|  | apoplexies |
|  | cerebro-vascular accidents |
|  | cerebral apoplexias |
|  | cerebral insults |
|  | hemisphere stroke |
|  | brain accident |
|  | cerebral stroke |
|  | cerebral strokes |
|  | stroke |
|  | syndrome after stroke |
|  | cerebrum vascular accident |
|  | brain vascular accidents |
|  | cerebral vascular accidents |
|  | attacks of the brain |
|  | vascular stroke |
|  | cerebrovascular stroke |
|  | cerebrovascular insults |
|  | stroke/cerebrovascular accidents |
|  | cerebro-vascular accident |
|  | cerebrovascular accident |
|  | cerebrum vascular accidents |
|  | cardiovascular stroke |
|  | brain ischemic attack |
|  | ischemic cerebral attack |
|  | brain arterial insufficiency |
|  | brain insult |
|  | stroke/cerebrovascular accident |
|  | cerebrovascular accidents |
|  | cerebral insult |
|  | apoplectic stroke |
|  | vascular strokes |
|  | CVA (Cerebral Vascular Accident) |
|  | brain attack |
|  | brain attacks |
|  | cerebral vascular accident |
|  | stroke syndrome |
|  | brain vascular accident |
|  | cerebrovascular apoplexy |
|  | cerebral apoplexy |
|  | brain ischemic attacks |
|  | hemisphere strokes |
|  | cardio-vascular stroke |
|  | apoplectic strokes |
|  | strokes |
|  | CVA (Cerebrovascular Accident) |
|  | ischemic cerebral attacks |
|  | brain accidents |
|  | cerebral apoplexies |
|  | cerebrovascular strokes |

---

|  |  |
| --- | --- |
| MeSH ID | D020521 |
|  | M0328070 |

---

|  |  |
| --- | --- |
| MeSH Heading | Stroke |

---
